# Integrative analysis of gene expression and histone modifications for *DES*, *DSP*, *GJA1* and *SMOC2* in adipose tissue reveals potential relationship to cardiometabolic health

**DOI:** 10.1186/s10020-025-01391-3

**Published:** 2025-11-22

**Authors:** Sadia Saeed, Anne Hoffmann, Stina Ingrid Alice Svensson, Tina Visnovska, Tobias Hagemann, Adhideb Ghosh, Christian Wolfrum, Akin Cayir, Tom Mala, Jon A. Kristinsson, Matthias Blüher, Tone Gretland Valderhaug, Yvonne Böttcher

**Affiliations:** 1 EpiGen, Medical Division, Akershus University Hospital, Lørenskog, Norway; 2 Department of Clinical Molecular Biology, EpiGen, Institute of Clinical Medicine, University of Oslo, Oslo, Norway; 3 Helmholtz Institute for Metabolic, Obesity and Vascular Research (HI-MAG) of the Helmholtz Zentrum München at the University of Leipzig and University Hospital, Leipzig, Germany; 4 Laboratory of Translational Nutrition Biology, Institute of Food, Nutrition and Health, ETH Zürich, Schwerzenbach, Switzerland; 5 Department of Endocrinology, Morbid Obesity and Preventive Medicine, Oslo University Hospital, Oslo, Norway; 6 Institute of Clinical Medicine, University of Oslo, Oslo, Norway; 7 Medical Department III – Endocrinology, Nephrology, Rheumatology, University of Leipzig Medical Center, University of Leipzig, Leipzig, Germany; 8 Department of Endocrinology, Akershus University Hospital, Lørenskog, Norway; 9 Department of Endocrinology, Obesity and Nutrition, Vestfold Hospital Trust, Tønsberg, Norway

**Keywords:** Obesity, Adipose tissue, Epigenetics, Gene expression, Cardiometabolic health

## Abstract

**Background:**

Adipose tissue influences cardiometabolic health through its endocrine activity and its role in regulating inflammation, lipid metabolism, and cardiovascular function. The expression of cardiac-associated genes within adipose tissue may reflect or contribute to cardiometabolic risk, yet this relationship remains poorly understood. This study investigates the expression profiles of the cardiac function associated genes *GJA1*, *DES*, *DSP* and *SMOC2* in human adipose tissue, and analyses their associations with cardiometabolic traits. Additionally, we explore epigenomic mechanisms that may underlie their differential gene expression.

**Methods:**

Expression profiling and functional enrichment analyses were conducted to identify depot-specific cardiac gene expression patterns. Quantitative PCR validated gene expression in paired subcutaneous (SAT) and omental visceral adipose tissue (OVAT) samples from 78 individuals with obesity. Gene expression was further validated in three independent cohorts (*N* = 1,548 total). Associations with clinical traits were assessed using Spearman correlations and multivariate linear regression, adjusted for age, sex, and BMI. Integration with transcriptomic and proteomic datasets publicly available from the Adipose Tissue Knowledge Portal was performed to strengthen clinical relevance. Epigenomic profiling using genome-wide ChIP-seq for histone marks (H3K4me3, H3K4me1, H3K27ac, H3K27me3) was conducted in paired SAT and OVAT samples from five individuals.

**Results:**

*DES*, *DSP*, *GJA1*, and *SMOC2* were significantly upregulated in OVAT compared to SAT. *DES*, *DSP*, and *SMOC2* showed consistent expression patterns across all cohorts, while *GJA1* exhibited context-dependent regulation. Gene expression in SAT was negatively correlated with cardiometabolic traits, including blood pressure, insulin resistance, and liver function markers. These associations were confirmed by regression analysis and supported by publicly available multi-omics data. Epigenetic analyses revealed OVAT-specific enrichment of active histone marks and reduced repressive marks, supporting higher differential transcriptional activity in OVAT.

**Conclusions:**

Depot-specific gene expression of *DES*, *DSP*, and *SMOC2* in adipose tissue is robustly linked to cardiometabolic traits and supported by distinct epigenetic landscapes in OVAT vs SAT, highlighting their potential as novel biomarkers for cardiometabolic health.

## Introduction

Obesity has emerged as a global public health concern, with more than 60% of the population in Europe being either overweight or obese (Boutari & Mantzoros 2022). Obesity is a major risk factor for a range of cardiometabolic disorders, including type 2 diabetes, dyslipidaemia and cardiovascular disease (Powell-Wiley et al. 2021). However, increasing evidence suggests that the distribution of adipose tissue, rather than total body fat alone, plays a critical role in determining the metabolic complications associated with obesity (Chait et al. 2020; Cornier et al. 2011; Frank et al. 2019). In particular, omental visceral adipose tissue (OVAT) is more strongly linked to adverse metabolic outcomes compared to subcutaneous adipose tissue (SAT), owing to its unique anatomical, cellular, and molecular characteristics (Ibrahim 2010; Neeland et al. 2013).

Recent studies by us and others, using epigenomic and transcriptomic profiling of various adipose tissue depots have revealed deeper insights into depot-specific signatures that may contribute to metabolic disease risk (Cannon et al. 2019; Divoux et al. 2020; Keller et al. 2017; Muller et al. 2024; Saeed et al. 2025; Schleinitz et al. 2020). In our recent work, we performed a genome-wide integrated analysis of chromatin accessibility (ATAC-seq) and gene expression profiles in paired SAT and OVAT samples from patients with obesity (Saeed et al. 2025). This analysis identified a set of genes including Gap junction alpha-1 (*GJA1*)*,* Desmin (*DES*), Desmoplakin (*DSP*), and SPARC-related modular calcium-binding protein 2 (*SMOC2*) that were higher expressed in OVAT as compared to SAT and enriched in pathways related to right ventricular cardiomyopathies and heart function (Saeed et al. 2025). Genetic mutations and aberrant functions of *DES*, *DSP*, *GJA1*, and *SMOC2* are implicated in cardiomyopathies through dysregulation of key cellular processes, including tissue remodelling, intercellular communication, and extracellular matrix regulation (Chen et al. 2024; Lee et al. 2023; Palatinus et al. 2023; Rui et al. 2022; Smith et al. 2020; Su et al. 2022; Tu et al. 2024). These processes are also central to adipose tissue biology and show depot-specific differences, with OVAT exhibiting greater remodelling, vascularization, and pro-fibrotic signatures than SAT (DeBari & Abbott 2020; Pellegrinelli et al. 2016; Sun et al. 2011). We therefore hypothesized that investigating the depot-specific expression of these genes, and their association with cardiometabolic traits, could provide novel insights into adipose remodelling and cardiometabolic risk.

Building upon these findings, we conducted a follow-up study in a larger in-house cohort of 78 individuals with paired SAT and OVAT samples to validate the depot-specific expression patterns of these cardiac-related genes and to explore their association with clinical variables of cardiometabolic health. Further, we successfully validated these findings across multiple other independent cohorts. We further investigated the chromatin landscape at these loci using in-house genome-wide histone modification data, focusing on key active and repressive marks such as H3K27 acetylation, H3K4me3, H3K4me1 and H3K27 trimethylation to better understand the regulatory underpinnings of their depot-specific expression.

## Methods

### Study design of the in-house cohort

Adipose tissue biopsies were collected from intra-individually matched samples of subcutaneous adipose tissue (SAT) and omental visceral adipose tissue (OVAT) during the initial phase of laparoscopic bariatric surgery in patients with obesity (*N* = 78; mean ± SD age 43 ± 10 years; Body mass index (BMI) 44.8 ± 6.4 kg/m^2^). A comprehensive set of anthropometric measurements and metabolic variables were available for all individuals (Table 1). For transcriptome analyses, biopsies were immediately snap-frozen on dry ice or in liquid nitrogen to prevent degradation and subsequently stored at −80 °C until further processing. The study protocols were approved by the Regional Ethics Committee for Health Region South-East Norway (2017/1528, 2013/2042, 489,516). All participants provided written informed consent prior to enrolment. The main characteristics of the study population are presented in Table 1.

**Table 1:** Clinical characteristics of the in-house cohort (data presented as mean ± standard deviation**)**

Traits	N	Mean	Std. Deviation
Age, yrs	78	43.41	10.97
Sex(male/female)	(23/55)		
Diabetes (y/n)	(12/66)		
Maximum weight, kg	72	132.34	27.68
Systolic BP	75	136.47	15.34
Diastolic BP	75	75.72	12.65
Waist, cm	77	125.13	14.42
Hip, cm	77	131.56	16.42
Neck, cm	77	41.08	4.46
Hight, cm	78	170.43	9.39
Weight, kg	78	131.07	24.45
Body mass Index (BMI), kg/m2	78	44.86	6.47
Waist-to-hip-ratio (WHR)	77	0.98	0.36
Fasting serum glucose (FSG)	78	5.96	1.31
C reactive protein (CRP)	78	10.32	8.52
Ferritin	78	125.03	80.34
Aspartate aminotransferase (ASAT), (U/L)	78	27.10	12.48
Alanine aminotransferase (ALAT), (U/L)	78	32.03	19.28
Alkaline phosphatase (ALP), (U/L)	78	80.72	22.14
Gamma-glutamyl transferase (GGT), (U/L)	78	46.14	60.60
Albumin (Alb)	78	42.38	2.34
Calcium (Ca)	78	2.35	0.07
Ca-alb-corr	78	2.33	0.06
Total Cholestrol	77	4.90	0.91
HDL cholesterol (mmol/l)	77	1.36	1.28
LDL cholesterol (mmol/l)	78	2.95	0.86
Triglycerides (mmol/l)	77	1.77	0.69
Parathyroid Hormone (PTH)	78	6.31	2.34
Heamoglobin (Hb)	78	17.46	18.95
Creatinine	78	66.97	12.86
Thyroid-stimulating hormone (TSH)	78	2.16	1.26
Free Thyroxine 4 (FT4)	78	15.54	2.32
F Thyroxine 3 (FT3)	78	5.17	0.69
Fast serum insuline (FSI)	74	221.91	242.35
C-peptid	76	1432.84	485.65
HOMA-IR	74	10.01	11.69
HbA1c IFCC	78	40.70	9.01

### Study design of the validation cohort

The validation cohorts were derived from the Leipzig Obesity Biobank (LOBB; https://www.helmholtz-munich.de/en/hi-mag/clinical-studies/leipzig-obesity-bio-bank-lobb), comprising intra-individually paired abdominal SAT and OVAT samples as reported in earlier studies (Saeed et al. 2025). All patients were extensively clinically phenotyped as previously described (Bluher 2020; Kloting et al. 2010). Adipose tissue samples were collected during elective laparoscopic abdominal surgeries, following established protocols (Langhardt et al. 2018). The cross-sectional cohort (CSC) consists of 1,480 individuals. Among them, 31 are classified as having no obesity (normal weight *N* = 12; or overweight: *N* = 19; 53% women; mean ± SD age 56.4 ± 13.3 years; BMI 25.5 ± 2.6 kg/m^2^), while the remaining 1,449 participants have obesity (71% women; mean age 46.9 ± 11.7 years; BMI 49.2 ± 8.3 kg/m^2^). The metabolically healthy/unhealthy obesity cohort (MHO/MUO; *N* = 73) includes 31 insulin-sensitive (IS) individuals (71% women; average age 38.8 ± 11.1 years; BMI 45.9 ± 6.9 kg/m^2^; fasting plasma glucose [FPG]: 5.2 ± 0.2 mmol/l; fasting plasma insulin [FPI]: 27.9 ± 13.5 pmol/l) and 42 insulin-resistant (IR) individuals (71% women; average age 47.2 ± 7.7 years; BMI 47.3 ± 8.1 kg/m^2^; FPG: 5.7 ± 0.3 mmol/l; FPI: 113.7 ± 45.7 pmol/l). The bariatric two-step surgery cohort (BSC) includes 65 individuals with severe obesity (66% women) who underwent weight-loss surgery at two separate time points. Their preoperative BMI at the first step surgery (sleeve gastrectomy) was 54.5 ± 9.3 kg/m^2^, and age was 44.1 ± 9.2 years. At the second surgery (gastric bypass), BMI averaged 40.9 ± 7.2 kg/m^2^, and age was 47.1 ± 9.9 years. Patients lost an average of 40.2 ± 21.2 kg, with only those losing more than 5 kg included. The LOBB study was approved by the Ethics Committee of the University of Leipzig (approval no: 159–12–21,052,012) and performed in accordance with the Declaration of Helsinki.

### Gene expression analysis by real-time quantitative PCR

For the in-house cohort, total RNA was isolated from SAT and OVAT using the RNeasy Plus Mini Kit (Qiagen), with protocol as previously described (Saeed et al. 2025). Complementary DNA (cDNA) was synthesized from 500 ng of total RNA using the High-Capacity cDNA Reverse Transcription Kit (Thermo Fisher Scientific). Subsequent quantitative PCR (qPCR) analyses were performed in duplicates. Gene expression was measured using the QuantStudio 7 Flex system (Thermo Fisher Scientific) with target-specific TaqMan Real-Time PCR assays (Thermo Fisher Scientific™). Relative gene expression levels were determined using the ΔCT method, normalizing to the housekeeping gene *PGK1* [2^-(CT_gene of interest – CT_*PGK1*)]. All TaqMan assays were commercially sourced (ThermoFisher Scientific™) with the following Assay IDs “*DES* (Hs00157258_m1), *DSP* (Hs00950591_m1), *GJA1* (Hs00748445_s1), *SMOC2* (Hs01591663_m1) and *PGK1* (Hs99999906_m1) as housekeeping control gene. Assays are designed to span exon–intron boundaries to avoid genomic DNA amplification.

### RNA sequencing analysis of the validation cohort

The library preparation and RNA sequencing (RNA-seq) data processing were conducted as previously described (Hagemann et al. 2023; Saeed et al. 2025). In brief, RNA was extracted using the SMARTseq protocol (Picelli et al. 2014) followed by single-end sequencing on a NovaSeq 6000. Reads were trimmed with Fastp (Chen et al. 2018) (v0.20.0), aligned to the human reference genome (GRCh38.p13; GENCODE release 32) (Frankish et al. 2019) and an expression quantification using Kallisto (Bray et al. 2016) (v0.48). The data were normalized using a weighted trimmed mean (TMM) and adjusted for age and sex.

### Chromatin immunoprecipitation and sequencing (ChIP-seq) of the discovery cohort

Genome-wide chromatin immunoprecipitation (ChIP) was performed for five intra-individually paired adipose tissue samples using our in-house protocol optimized for frozen human adipose tissue, specifically tailored for histone modification ChIP, as previously described (Cayir et al. 2024). Briefly, approximately 100 mg of frozen SAT or OVAT was crosslinked with 1% formaldehyde, quenched with glycine, and homogenized. Chromatin was then isolated and sheared using a Bioruptor Plus sonicator (Diagenode, UCD-300) to obtain DNA fragments ranging from 200–600 base pairs. After pre-clearing, the chromatin was incubated with Protein A Dynabeads (Thermo Fisher Scientific) conjugated to histone modification-specific antibodies: 2.5 µg each of H3K4me3 (C15410003, Diagenode), H3K27ac (C15410174, Diagenode), and H3K27me3 (C15410195, Diagenode), and 1 µg of H3K4me1 (C15410037, Diagenode). Following immunoprecipitation, sequential washes were performed, followed by elution, reverse crosslinking, and DNA purification. ChIP libraries were then prepared using the ThruPLEX DNA-Seq kit (Takara Bio USA) and sequenced as 150 bp paired-end reads on the HiSeq 4000 platform (Illumina) at the Norwegian Sequencing Centre, Oslo University Hospital.

### ChIP-seq data processing and peak calling

Sequencing data quality was assessed using FastQC (Andrews n.d.), followed by read trimming with Trimmomatic (Bolger et al. 2014) to remove low-quality reads and adapter sequences. Trimmed reads were aligned to the human genome (hg38) with Bowtie2 (Langmead et al. 2019) and processed using Picard (Picard tools n.d.: http://broadinstitute.github.io/picard/) and Samtools. (Li et al. 2009) to remove duplicates and low-quality or mitochondrial reads. Peaks were called with MACS2 (Zhang et al. 2008), filtered using ENCODE blacklists, and converted to bigWig files. Peaks from all samples were merged and filtered to retain only those present in at least two samples. Read counts for called peaks were obtained using featureCounts (Liao et al. 2014). Signal intensity at called peaks was calculated as BPM (bins per million reads). This analysis was performed on the TSD (Tjeneste for Sensitive Data) facilities, developed by the IT-Department (USIT) at the University of Oslo. Histone modifications signal tracks were visualized using the Integrative Genomic Viewer (IGV) browser (Robinson et al. 2011).

### Statistical analyses

All statistical analyses of the discovery cohort were performed using IBM SPSS Statistics version 30, GraphPad Prism version 10, and R version 4.2.3. The paired t test was used to assess depot-specific differences in target genes between the two adipose tissue depots. For the correlation with clinical variables in the discovery cohort, the distribution of all variables was assessed using the Kolmogorov–Smirnov test and visual inspection of histograms. Variables not normally distributed were log-transformed to approximate a normal distribution. Bivariate Spearman’s correlation was used to explore associations between gene expression and clinical variables. Linear regression analyses were applied to further examine these relationships while adjusting for potential confounders such as age, gender, and BMI, where applicable. A P value of less than 0.05 was considered significant. In the validation cohorts, group comparisons were performed using Wilcoxon tests implemented via the rstatix R package (v0.7.2). Statistical significance was assessed with P values adjusted for multiple testing using the Bonferroni correction method. Analyses were conducted in R 4.5.0.

## Results

### Depot-specific expression of cardiac-related genes in human adipose tissue

**Fig. 1:**
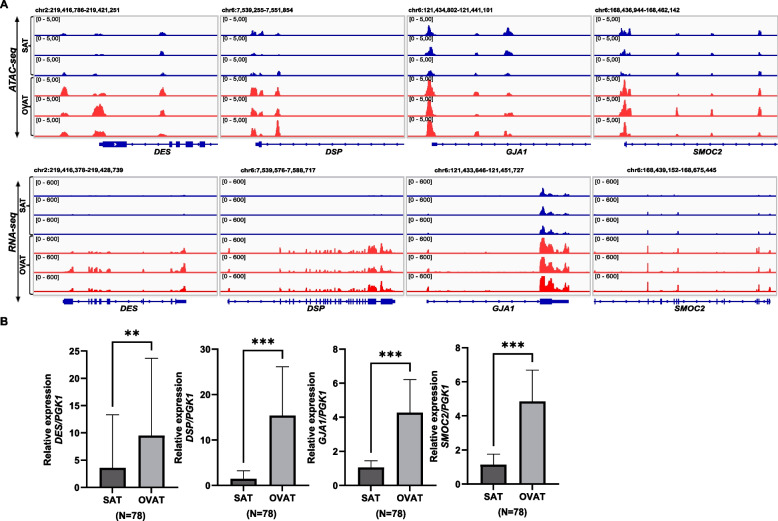
Depot-specific expression of cardiac-related genes in the in-house cohort. **A** IGV browser view of the ATAC-seq and RNA-seq data showing differential accessibility and gene expression along the studied genes loci in both SAT (blue) and OVAT (red) samples. **B** Barplots showing relative expression levels of *DES*, *DSP*, *GJA1*, and *SMOC2* in paired subcutaneous adipose tissue (SAT) and omental visceral adipose tissue (OVAT) samples from 78 patients. Data are presented as mean ± standard deviation. A paired t-test was used to assess statistical significance between depots

Our previously published integrated ATAC-seq and RNA-seq analysis of paired SAT and OVAT identified four cardiac function related genes *GJA1*, *DES*, *DSP*, and *SMOC2* that showed higher chromatin accessibility and expression in OVAT compared to SAT (Fig. 1A). In the current study, we validated the depot-specific gene expression of *GJA1*, *DES*, *DSP*, and *SMOC2* in intra-individually paired SAT and OVAT samples from 78 individuals with obesity from our in-house cohort using quantitative PCR. All four genes showed significantly higher gene expression (*DES*; *p* value < 0.004, *DSP*, *GJA1* and *SMOC2*; *p* value < 0.001) in OVAT compared to SAT (Fig. 1B), consistent with our initial genome-wide analysis (Saeed et al. 2025).

### Independent validation of depot-specific expression patterns of cardiac-related genes

**Fig. 2:**
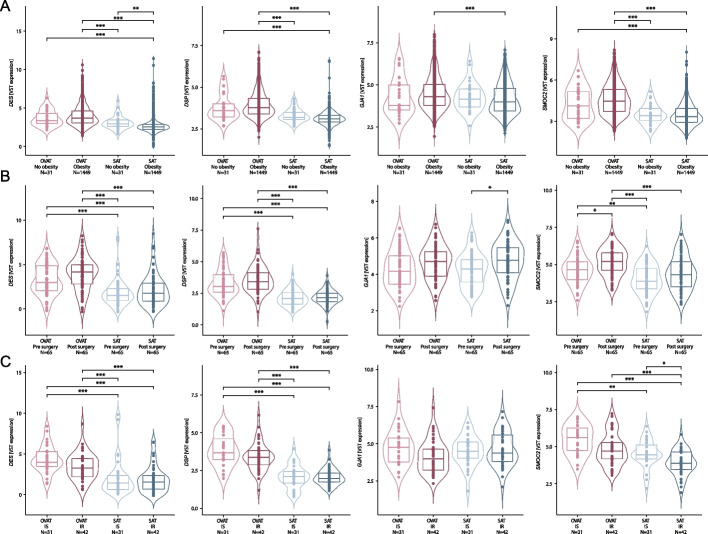
Depot-specific expression of cardiac-related genes in independent human cohorts. Gene expression comparisons of the *DES*, *DSP*, *GJA1*, and *SMOC2* between OVAT and SAT within (**A**) the cross-sectional cohort (no obesity: *N* = 31; obesity: *N* = 1.449), (**B**) the two-step bariatric surgery cohort (pre and post-surgery: *N* = 65) and within (**C**) the metabolically healthy versus unhealthy obesity cohort (insulin sensitive: *N* = 31, insulin resistant: *N* = 42) of LOBB. Gene expression data are based on TMM-normalized RNA sequencing data, adjusted for sex and age. Statistical significance was assessed using Wilcoxon pairwise comparisons with Bonferroni correction

To further validate our findings of depot-specific gene expression patterns of these four cardiac function-associated genes, we analysed their expression levels across three independent validation cohorts from the LOBB (details see methods). In the large cross-sectional cohort (CSC; *N* = 1,480), all four genes consistently showed significantly higher expression in OVAT compared to SAT in individuals with obesity (BMI > 30 kg/m^2^, *N* = 1,449, Fig. 2A). Moreover, although non-significant, we observed the same effect direction for *DES, DSP* and *SMOC2* among individuals without obesity (*N* = 31) (Fig. 2A). In a second cohort consisting of individuals with morbid obesity undergoing a two-step bariatric surgery (BSC; *N* = 65) aimed at weight loss, *DES*, *DSP*, and *SMOC2* were successfully validated with significant higher expression in OVAT compared to SAT, while *GJA1* did not reach statistical significance (Fig. 2B). *GJA1* in SAT and *SMOC2* in OVAT also exhibit significantly increased gene expression levels following weight loss. Although not statistically significant, this trend is observable across all other tissues and genes as well. In line with this, in a third cohort stratified by metabolic health status among individuals with obesity (MHO/MUO), *DES*, *DSP*, and *SMOC2* again showed significantly higher gene expression in OVAT compared to SAT. Although only *SMOC2* in SAT reached statistical significance, lower gene expression across all genes was observed in individuals with insulin resistance (IR; *N* = 42) compared to their insulin-sensitive (IS; *N* = 31) counterparts, with this pattern being more pronounced in OVAT (Fig. 2C). These findings support a consistent depot-specific expression of *DES*, *DSP*, and *SMOC2* across multiple cohorts, with higher gene expression levels in OVAT compared to SAT.

### Association between depot-specific gene expression and cardiometabolic traits

**Fig. 3:**
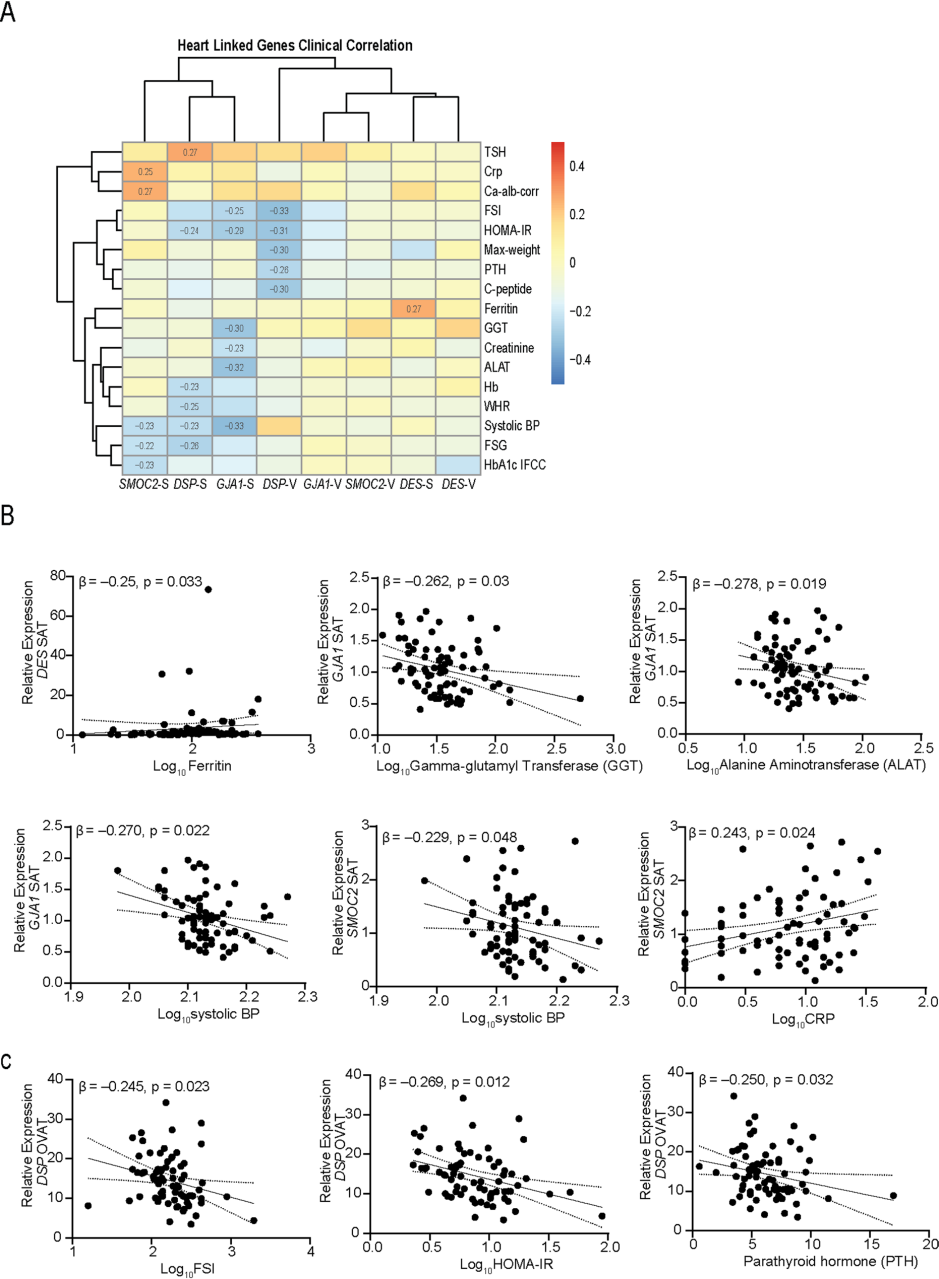
Correlation of cardiac-related genes expression with clinical and metabolic parameters. **A** Heatmap showing bivariate Spearman rank correlation between clinical variables and relative expression of *DES, DSP, GJA1* and *SMOC2* in SAT and OVAT. Only statistically significant correlations (p < 0.05) are annotated with corresponding r values. Hierarchical clustering was applied to both genes and clinical traits to identify patterns of association. Colour scale represents correlation strength and direction (red = positive, blue = negative). **B** Scatter plots depicting individual linear regression models for selected gene-clinical trait associations, adjusted for age, sex, and BMI. The β coefficient and p-value for each association are shown. All models demonstrate significant associations (*p* < 0.05)

To assess the potential clinical relevance for cardiometabolic health, we examined the correlation between gene expression levels and a range of metabolic and cardiometabolic variables (Table 1) using Bivariate Spearman’s rank correlation within the in-house cohort. We observed a consistent negative correlation of gene expression of *GJA1*, *DSP*, and *SMOC2* in SAT with systolic blood pressure (*p* < 0.05), underlining a potential link to cardiovascular function (Fig. 3A). In addition to blood pressure, significant correlations (P < 0.05) were observed with key metabolic and anthropometric traits. In the subcutaneous adipose tissue depot, gene expression of *DSP* and *GJA1* was negatively correlated with Homeostatic Model Assessment for Insulin Resistance (HOMA-IR), indicating at a potential role in insulin sensitivity. *GJA1* and *SMOC2* were also inversely associated with fasting serum insulin levels, while *DSP* and *SMOC2* gene expression showed negative correlation with fasting serum glucose (FSG), reinforcing a potential link to glucose metabolism (Fig. 3A).

Each gene also displayed distinct clinical associations. *DSP* expression in SAT correlated negatively with haemoglobin (Hb) and waist-to-hip ratio (WHR), and in OVAT, with maximum weight, parathyroid hormone (PTH), and C-peptide levels. Additionally, *DSP* in SAT showed a positive correlation with thyroid-stimulating hormone (TSH). *GJA1* expression in SAT was negatively correlated with liver and kidney function markers, including alanine aminotransferase (ALAT), creatinine, and gamma-glutamyl transferase (GGT). *SMOC2* in SAT showed positive associations with C-reactive protein (CRP) and calcium–albumin correction. *DES* expression in SAT was positively correlated with ferritin levels (Fig. 3A).

In linear regression analyses adjusted for age, sex, and BMI, several of these correlations remained significant (Fig. 3B-C). *DSP* expression in OVAT remained inversely associated with fasting serum insulin (β = –0.269, *p* = 0.023), HOMA-IR, and PTH (Fig. 3C). For *GJA1*, SAT expression remained significantly associated with systolic blood pressure (β = –0.270, *p* = 0.022) and GGT (β = –0.262, *p* = 0.03). *SMOC2* expression was independently linked to CRP (β = 0.243, *p* = 0.024), systolic blood pressure (β = –0.229, *p* = 0.048). Additionally, after adjusting for age and BMI only, *DES* expression in SAT remained associated with ferritin (β = –0.25, *p* = 0.033), and *GJA1* in SAT retained association with ALAT (β = –0.278, *p* = 0.019).

### Cross-dataset clinical associations: integration with proteomic and transcriptomic data

**Fig. 4:**
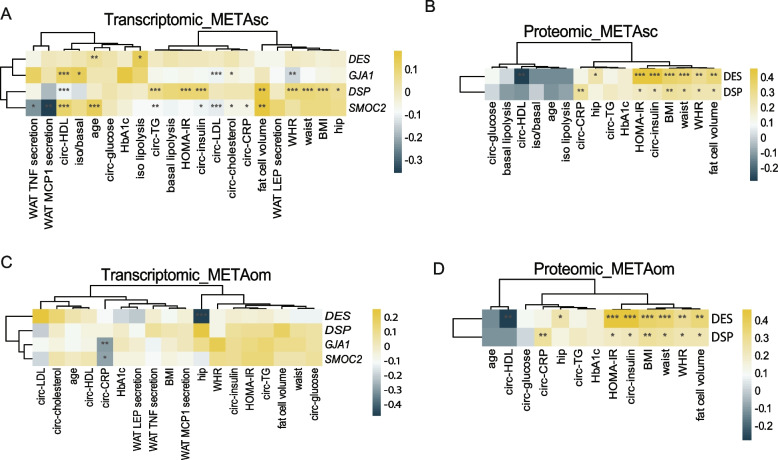
Correlation of transcriptomic and proteomic expression levels with clinical traits using the Adipose Tissue Knowledge Portal. Heatmaps display the correlation between expression levels (RNA and protein) and clinical traits (**A**-**D**) using publicly available datasets of subcutaneous adipose tissue (sc; A-B) and omental visceral adipose tissue (om; **C**-**D**). The "clinical" module of the AT Knowledge Portal was used to assess these associations

To further explore the clinical relevance of our candidate genes, we leveraged the Clinical Module of the Adipose Tissue Knowledge Portal (https://adiposetissue.org/) (Zhong et al. 2025) which enables integrated transcriptomic and phenotypic correlations across different human cohorts of SAT and OVAT. This analysis enabled a comprehensive evaluation of transcriptomic and proteomic associations for *GJA1*, *DSP*, *DES*, and *SMOC2* with a broad range of clinical parameters (Fig. 4).

When comparing association of transcriptomic data with clinical variables, we observed that significant associations were largely restricted to SAT in both our cohort and the adiposetissue.org datasets (Zhong et al. 2025). For example, *GJA1*, *SMOC2*, and *DSP* expression in SAT correlates with cardiometabolic traits such as HbA1c, HOMA-IR, and inflammatory markers such as CRP. Despite some cohort-specific differences in effect sizes and direction of correlation, the recurrence of these associations in SAT across datasets implies a potential broader clinical relevance.

Additionally, proteomic data for *DES* and *DSP* showed strong associations with key markers of metabolic dysfunction including WHR, waist circumference, BMI, circulating insulin, HOMA-IR, and CRP further supporting their potential clinical significance.

Taken together, the cross-cohort transcriptomic overlaps, complemented by proteomic associations from adiposetissue.org, suggest that depot-specific expression of *DES, DSP*, *GJA1*, and *SMOC2* is linked to cardiometabolic traits, supporting their potential relevance as biomarkers or modulators of adipose tissue related metabolic health.

### Epigenomic profiling reveals depot-specific regulatory landscapes

To explore the regulatory mechanisms underlying the differential gene expression of *GJA1*, *DES*, *DSP*, and *SMOC2*, we analysed chromatin states around these genes’ loci using our in-house ChIP-seq data for four active and repressive histone modifications: H3K4me3, H3K4me1, H3K27ac, and H3K27me3. ChIP-seq data on histone modifications was generated under the same conditions as our expression analysis, using paired SAT and OVAT samples from five patients with obesity. The histone marks analysed here highlight key regulatory elements; H3K4me3 and H3K4me1 are associated with promoters and enhancers, while H3K27ac and H3K27me3 reflect active and repressed chromatin states, respectively.

**Fig. 5:**
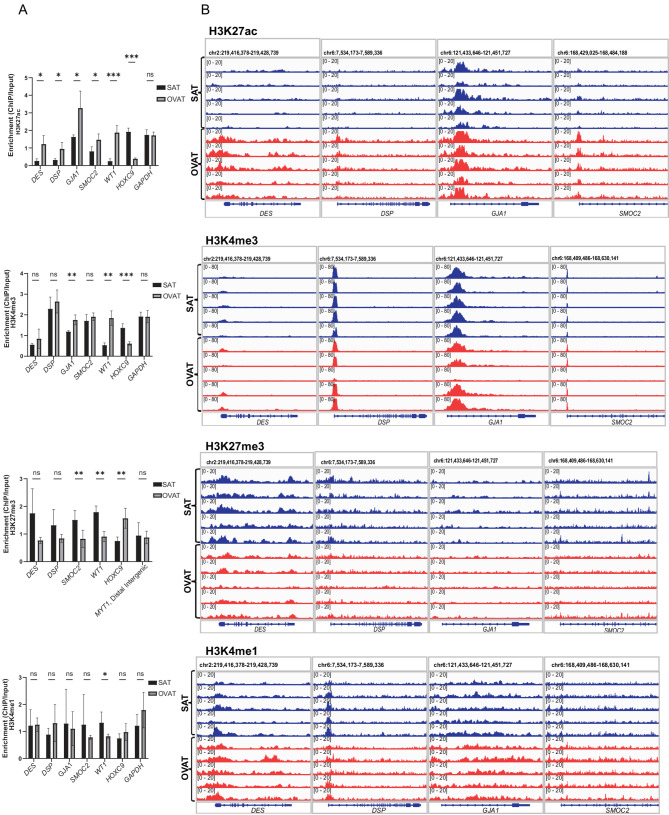
**A** Bar plots representing ChIP enrichment over Input for four histone modifications (H3K27ac, H3K4me3, H3K4me1, and H3K27me3) at the promoter regions of the four cardiac-related genes, along with three control genes: WT1 (OVAT-specific), HOXC9 (SAT-specific), and GAPDH (positive control for both tissues). ChIP signal intensity is quantified as BPM (bins per million) values. **B** Integrative Genomics Viewer (IGV) screenshots showing representative ChIP-seq signal tracks for the same histone modifications across the promoter regions of the four selected genes

In addition to the cardiac-related genes, we also included *WT1*, *HOXC9*, and *GAPDH* as reference controls, while *MYT1* gene (distal intergenic region) was used as positive control for H3K27me3 only. *WT1* and *HOXC9* were selected based on prior studies reporting depot-specific gene expression, with *WT1* enriched in OVAT and *HOXC9* preferentially expressed in SAT (Brune et al. 2016; Chau et al. 2014; Chau & Hastie 2015; Dommel et al. 2020; Kirschner & Scholz 2022). The inclusion of these reference genes provided an internal validation of our ChIP-seq approach. As expected, we observed higher levels of active histone marks (H3K27ac, H3K4me3) at the *WT1* locus in OVAT and at the *HOXC9* locus in SAT, consistent with their known depot-specific expression, while repressive marks showed the opposite pattern (Fig. 5A). In contrast, the housekeeping gene *GAPDH* displayed no depot-specific differences in histone modifications, confirming the specificity of the observed depot-dependent chromatin changes.

For *GJA1*, *DES*, *DSP*, and *SMOC2*, we observed an enrichment of active histone marks (H3K4me3, H3K27ac) in both SAT and OVAT, with higher levels consistently observed in OVAT (Fig. 5A), which is in line with higher gene expression in OVAT. Notably, the repressive mark H3K27me3 was reduced in OVAT across three of the four genes and completely absent at the *GJA1* locus in both depots, suggesting an open chromatin configuration conducive for gene activation. IGV browser tracks further illustrated this depot-specific enrichment of activating marks, particularly H3K27ac, at promoter regions (Fig. 5B) showing clearly higher peaks in OVAT compared to SAT. These epigenetic distinctions likely underpin the depot-specific gene expression patterns observed for these cardiac-related genes.

## Discussion

This study identifies and characterizes the depot-specific gene expression of the four genes (*GJA1*, *DES*, *DSP*, and *SMOC2*) across intra-individually paired samples of human subcutaneous (SAT) and omental visceral adipose tissue (OVAT), revealing their potential relevance to cardiometabolic health. Through multi-cohort validation, integrative correlation analyses, and epigenomic profiling of histone modifications, we provide robust evidence linking these genes to cardiometabolic traits and distinct adipose tissue regulatory landscapes.

We first demonstrate that all four genes exhibit significantly higher gene expression in OVAT compared to SAT, a pattern consistently replicated across several independent human cohorts. Interestingly, within the same adipose tissue depot, all genes exhibit a tendency for lower gene expression among individuals with insulin resistance compared to insulin sensitive subjects, which may hint at a potential role in insulin metabolism. In general, these findings underline the functional heterogeneity of the human adipose tissue depots, which is important in understanding its metabolic implications in the aetiology of metabolic disorders.

Further, in correlation analyses with clinical traits related to anthropometry and metabolism, we observed that several associations of genes expression and cardiometabolic parameters remained significant even after adjusting for age, sex, and BMI. Specifically, *DSP* expression in OVAT was inversely associated with fasting insulin, HOMA-IR, and parathyroid hormone (PTH). In SAT, *GJA1* expression showed negative associations with GGT, ALAT, and systolic blood pressure, while *SMOC2* expression was positively correlated with CRP and inversely with systolic blood pressure. These associations indicate that these genes might be implicated in both vascular and metabolic regulation, supporting prior studies suggesting that OVAT contributes more directly to cardiometabolic risk (Chiba et al. 2007; Despres 2012). Importantly, we corroborated these associations through independent transcriptomic and proteomic analyses of datasets originating from the Adipose Tissue Knowledge Portal (Zhong et al. 2025). Hence, the cross-dataset consistency in gene-clinical trait correlations further strengthens and underlines their potential relevance as biomarkers or important players in cardiometabolic health. These genes are described for being important for cardiac function and related cardiomyopathies directly linked to gene defects/mutations or genetic variation within these genes (*DES*, *DSP*, *GJA1*)(Brandao et al. 2023; Su et al. 2022; Van Norstrand et al. 2012). However, we find a range of correlations with clinical traits that may be linked to cardiometabolic status that is potentially independent from more direct genetic effects of these genes on cardiac structure and function. Taken together, we discover a novel avenue of how gene expression of these genes within adipose tissue might be connected to cardiometabolic health.

Moreover, we have here presented ChIP-seq profiling of histone modifications in paired samples of human SAT and OVAT revealing depot-specific differences in epigenetic regulation. Active histone marks (H3K4me3, H3K27ac) were enriched at the promoters of these genes, particularly in OVAT, whereas repressive H3K27me3 signals were more prevalent in SAT. Notably, the absence of H3K27me3 at the *GJA1* locus in both depots suggests its poised regulatory potential. These findings support the hypothesis that epigenetic landscapes underlie depot-specific gene expression and may be potential modifiable targets for metabolic disease intervention (Bradford et al. 2019; Roadmap Epigenomics et al. 2015). This kind of ChIP-seq data from paired samples of SAT and OVAT is, to our knowledge, not yet available from any other public source and therefore one of the unique resources generated in this study from a challenging human heterogeneous tissue like adipose. Nevertheless, the ChIP-seq analysis was conducted in a small number of paired SAT and OVAT samples (*N* = 5). While this dataset provides valuable depot-specific insights, the limited sample size constrains the generalizability of these findings and highlights the need for replication in larger cohorts.

While our findings emphasize the upregulation of *GJA1*, *DES*, *DSP*, and *SMOC2* in OVAT linked to greater cardiometabolic risk, it is also plausible that the downregulation of these genes in SAT may itself contribute to cardiovascular disadvantage that may contribute to worsened cardiometabolic health. Given that these genes are critical for cardiac structure and function (McLendon & Robbins 2011; Palatinus et al. 2023; Smith et al. 2020; Williams et al. 2018), their reduced expression in SAT could reflect a loss of protective signalling or structural integrity within this depot. Our data also raise the possibility that lower expression of these genes in SAT compared to OVAT may represent an underrecognized contributor to cardiovascular vulnerability. However, these findings are exploratory in nature and require validation through targeted functional studies to further investigate this along with potential causative mechanistic implications. In addition, the interpretation of these findings is limited by the composition of our study cohorts. The in-house cohort included only individuals with obesity, without normal-weight controls, which restricts our ability to determine whether the observed gene expression patterns are obesity-specific. Moreover, in the CSC of the LOBB cohort, the markedly imbalanced group sizes between obese and non-obese individuals (*N* = 1,449 vs. *N* = 31), together with the relatively high proportion of overweight participants (*N* = 19) in the non-obese group, may have influenced the observed results. Therefore, our findings should be regarded as exploratory. The causal relationships between gene expression changes and phenotypic outcomes have not yet been experimentally validated. Moreover, future studies should also delineate not only the detrimental effects of OVAT in metabolic diseases, but also potential protective mechanisms of SAT gene expression in maintaining systemic metabolic homeostasis.

## Conclusions

In conclusion, this study uncovers a set of cardiac-associated genes enriched in OVAT with consistent links to insulin sensitivity inflammation, and cardiovascular traits. The integration of gene expression, epigenomic, and clinical data provides a multidimensional view of adipose tissue function and highlights novel targets for further exploration in the context of obesity and its related co morbidities like cardiovascular disease. While these findings provide important insights into depot-specific gene regulation, their interpretation is limited by cohort composition and sample size. Future studies including normal-weight controls and larger epigenomic datasets are warranted to strengthen causal inference and translational potential.

## Data Availability

All the data associated with the current study is included in this article.

## Abbreviations

SAT: Subcutaneous adipose tissue
OVAT: Omental visceral adipose tissue
*GJA1*: Gap junction alpha-1
*DES*: Desmin
*DSP*: Desmoplakin
*SMOC2*: SPARC-related modular calcium-binding protein 2
ChIP: Chromatin immunoprecipitation
LOBB: The Leipzig Obesity Biobank
CSC: Cross-sectional cohort
MHO/MUO: The metabolically healthy / unhealthy obesity
BSC: Bariatric two-step surgery cohort
BMI: Body mass index
IS: Insulin-sensitive
IR: Insulin-resistant
HOMA-IR: Homeostatic Model Assessment for Insulin Resistance
FSG: Fasting serum glucose
FSI: Fasting serum insulin
Hb: Haemoglobin
WHR: Waist-to-hip ratio
PTH: Parathyroid hormone
TSH: Thyroid-stimulating hormone
ALAT: Alanine Aminotransferase
GGT: Gamma-glutamyl transferase
CRP: C-reactive protein
